# Community Colleges and Public Health: An Integral Part of the Continuum of Education for Public Health

**DOI:** 10.3389/fpubh.2014.00226

**Published:** 2014-11-05

**Authors:** Richard Kenneth Riegelman, Cynthia Wilson

**Affiliations:** ^1^Epidemiology and Biostatistics, Milken Institute School of Public Health, The George Washington University, Washington, DC, USA; ^2^Vice President Learning and Research, League for Innovation in the Community College, Chandler, AZ, USA

**Keywords:** undergraduate public health education, community colleges, continuum of public health education, health education, health administration, environmental health sciences, health navigator, community health workers

## Introduction and Background

Undergraduate education for public health has grown rapidly in the last decade since the Institute of Medicine recommended that “…all undergraduates should have access to education in public health.” Despite the growth of undergraduate education for public health in 4-year institutions, public health education in community colleges is at an early stage of development. In a comprehensive 2011–12 web-based catalog search of community colleges, only seven associate degree programs in public health or related fields could be identified (1, 2).

Public health organizations are encouraging growth of education for public health in community colleges as well as 4-year colleges. The American Public Health Association has endorsed undergraduate public health education at both community colleges and 4-year colleges (3). Healthy People 2020 includes objectives to substantially increase the number of community colleges as well as 4-year institutions offering undergraduate public health education (4).

As part of the Framing the Future Task Force, convened by the Association of Schools and Programs of Public Health, the Community Colleges and Public Health (CC&PH) project, has been developed and co-sponsored by the League for Innovation in the Community College (the League), which represents over 800 of the 1100 community colleges. The CC&PH is co-chaired by the two authors of this article. The mission of the CC&PH project is to fully include community colleges in the continuum of public health education. The Community Colleges and Public Health Report (5) is expected to be a component of the final report of the Framing the Future Task Force (6).

The CC&PH project has included two phases, a first phase consisting of an Expert Panel, which developed a series of Foundation and Consensus Statements that reflected what public health and community college educational organizations could do together (5). The second phase, recommended by the Expert Panel, focused on development of “prototype curricular models” designed for associate degrees and academic certificate programs in community colleges. Two basic models, (1) Public Health: Generalist and Specialization and (2) Health Navigator,^1^ were chosen after consultations with community colleges, project and Task Force leadership, and public health practice organizations (ASTHO and NACCHO), as well as academic associations in disciplines, which offer related bachelor’s degree programs (SOPHE, AUPHA, and AEHAP^2^).

The CC&PH report recommends that Public Health associate degrees should be built on fundamental skills including writing, oral communications, and quantitative skills consistent with the Association of American Colleges and Universities (AAC&U) LEAP initiative (7) and VALUE Rubrics (8). Associate degrees and academic certificate programs are also encouraged to incorporate ASPPH Undergraduate Public Health Learning Outcomes. (9).

The Community Colleges and Public Health Project report recommends academic programs in Public Health: Generalist and Specializations designed for transfer to bachelor’s degree programs in general public health, health education, health administration, or environmental health. It also recommends Health Navigator academic certificate and associate degree programs. The CC&PH report also recommends specific courses and provides recommended content outlines: http://www.league.org/league/projects/ccph/.

The remainder of this article summarizes the two prototype curricular models and discusses next steps in implementation.

## Public Health: Generalist and Specialization – Recommendations

These associate degree programs are designed as transfer degrees to enable students to enroll in the rapidly growing bachelor’s degree programs in public health (generalist), as well as the specializations of health education, health administration, and environmental health. Specializations have been designed in collaboration with SOPHE, AUPHA, and AEHAP. The involvement of these organizations should facilitate but not ensure transferability of the recommended course work to bachelor’s degree programs.

Associate degrees designed for transfer to a bachelor’s degree are encouraged to include 30 semester credit hours of public health related course work, including experiential practice-based learning, out of a 60 semester credit hour degree program. To optimize student transfer and student mobility to 4-year programs, the associate degree programs are encouraged to teach associate degree courses that meet baccalaureate degree expectations.

Public Health: Generalist and Specialization – the 30 semester hours of public health and related coursework in a 60 semester credit hour associate degree program should include the following.

### Foundational

Human Health/Personal Health and Wellness – 3 semester credit hours.
Students need an introduction to the underlying science of human health and disease including opportunities for promoting and protecting health across the lifespan and including principles of population health and determinants of health.

### Public health core

Overview of Public Health – 3 semester credit hours.
Health Communications – 3 semester credit hours.
Coursework should be consistent with the ASPPH Undergraduate Baccalaureate Critical Component Elements (CCEs) Report.

### Required courses

Health Education – 3 semester credit hours.
Health Administration – 3 semester credit hours.
Environmental Health – 3 semester credit hours.

Alternatively, nine semester credit hours in one of these three disciplines may be substituted utilizing content outlines for three coordinated courses developed in collaboration with the corresponding academic association and designed for transfer to a bachelor’s degree. Detailed content outlines developed in collaboration with SOPHE, AUPHA, and AEHAP are available at http://www.league.org/league/projects/ccph/.

### Experiential learning

Experiential learning – 3 semester credit hours.
Supervised curriculum with learning outcomes and opportunities for reflection.

### Electives

Six to nine credit hours including a course in Public Health Preparedness as well as such courses as Prevention and Community Health, Health and Diversity, Global Health, etc.

Together the foundation and core public health courses should provide an introduction to at least the following CCEs: introduction to the biological and life sciences and the concepts of health and disease; overview of public health; health communications; identifying and addressing population health challenges; determinants of health; and overview of the health system (10): http://www.aspph.org/educate/models/undergraduate-baccalaureate-cce-report/.

Specific bachelor’s degree programs may require additional course work. Students are advised to consult the specific requirements of the bachelor’s degree program to which they wish to transfer. The development of the recommended content outlines in collaboration with SOPHE, AUPHA, and AEHAP is expected to facilitate transfer of these courses to bachelor’s degree programs. Community colleges may want to develop articulation agreements with bachelor’s degree programs to ensure transferability.

## Health Navigator Programs – Recommendations

Health Navigator associate degree programs are designed primarily as applied degrees intended to respond to the rapidly growing job market for assisting individuals to navigate the increasingly complex public health, health care, and health insurance systems. The rapid increase in employment opportunities as Community Health Workers, patient care navigators, and health insurance navigators increasingly requires professionalization of the field.

In addition to employment opportunities immediately upon graduation, the Society for Public Health Education (SOPHE) has endorsed the development of Health Navigator associate degrees designed for transfer to a bachelor’s degree in Health Education. SOPHE has encouraged the development of transfer programs and articulation of degrees with the large number of bachelor’s degree programs in Health Education as part of the continuum of public health education.

Academic certificate programs may also be offered for those with the work experience or previous academic degrees. In addition, those enrolled in other associate degree programs in the health professions and human services fields may find a Health Navigator academic certificate program is a valuable addition to their degree. All academic certificate programs should include the four required Health Navigator courses described below using the content outlines included in the CC&PH report: http://www.league.org/league/projects/ccph/.

The recommendations for Health Navigator degree and academic certificate programs are intentionally designed to provide community colleges with flexibility to meet local needs and state certificate requirements where applicable. In addition, the electives offered by each institution may be tailored to the needs of the local workforce.

New funding mechanisms as part of Medicaid and the Medicare 30-day hospital re-admission policy, as well as the Affordable Care Act have dramatically increased the interest in developing the types of paid positions requiring academic Health Navigator education. The Labor Department estimates that the positions for Community Health Workers, the only Health Navigators job classification tracked by the Labor Department, will increase over 20% by 2022 (11).

Thirty public health and related semester credit hours are recommended as part of a 60 semester credit hour Health Navigator associate degree program. These should incorporate many of the Association of Schools and Programs of Public Health’s Undergraduate Baccalaureate CCEs. Additional general education courses including those that focus on written and oral communication skills and basic quantitative skills are key to student success.

### Foundational

Human Health/Personal Health and Wellness – 3 semester credit hours.

### Public health core

Overview of Public Health – 3 semester credit hours.
Health Communications – 3 semester credit hours.

### Required courses

Accessing and Analyzing Health Information – including searching for health information and evaluating its validity – 3 semester credit hours.
Prevention and Community Health – 3 semester credit hours.
Health Care Delivery – 3 semester credit hours.
Health Insurance – 3 semester credit hours.

### Experiential learning

Experiential Learning in Community Health, Health Care Delivery and/or Health Insurance – 3 semester credit hours.
The experience needs to address outreach methods and strategies; client and community assessment; support, advocacy and coordination of care for clients; and community capacity building, including supervised curriculum with learning outcomes and opportunities for reflection.

### Electives

Six semester credit hours addressing state and local regulations and job markets. These may include specific diseases such as diabetes, cancer, cardiovascular disease, HIV, defined populations such as the elderly, maternal, and child, and/or population issues such as health and diversity, as well as global health. A public health preparedness course should also be offered.
Additional courses, Introduction to Health Education and the Public Health Advocacy and Leadership in Action courses, designed by SOPHE for transfer to bachelor’s degree programs in Health Education should be taken by students who intend to transfer to a Health Education bachelor’s degree program. Content outlines for these courses can be found at http://www.league.org/league/projects/ccph/.

Health navigator education is in the early phase of development and is likely to undergo rapid growth and change. Growth is likely to be very rapid in the remaining years of this decade as the need for team-based delivery of health services gains momentum and the new funding mechanisms for paid positions become established.

Note that the Foundation course and the two core public health courses are the same for all associate degree programs recommended in the CC&PH report. This should allow students enrolled in community colleges with multiple public health related programs to initially pursue foundational and core public health courses before selecting between available associate degree offerings.

The recommended programs are not the only possible public health programs that may be offered in community colleges. For instance, the CC&PH report recognizes the potential for health information management programs with a public health focus. Community colleges may identify additional public health programs that meet their mission and the needs of the local workforce. In addition, community colleges may play an important role in the continuing education of the public health workforce, especially entry-level employees and those without prior education for public health.

## Next Steps as Recommended in the Community Colleges and Public Heath Report

The commitment of public health education and practice organizations provides opportunities to fully develop the continuum of public health education from community colleges through graduate education. Based on the extraordinarily diverse student body represented in many community colleges, this collaboration also provides unique opportunities to develop a diverse public health workforce, which reflects the current and future populations of the United States.

The principles outlined in the report should help local and state health departments and other employers address the educational needs and aspirations of the public health workforce of the future. These programs should be accessible to entry-level employees.

As a key component of the next steps, the League for Innovation in the Community College seeks to work closely with the national practice and academic organizations collaborating in this report to identify funding to support demonstration projects. These demonstration projects should provide opportunities for community colleges to fully develop curricula complete with learning outcomes/competencies, assessments tools, etc. The League also intends to develop community college recognition awards for excellence in planning education for public health.

The ongoing collaboration of public health academic and professional organizations with the League for Innovation in the Community College bodes well for the future of community college education for public health.

## Conflict of Interest Statement

The authors declare that the research was conducted in the absence of any commercial or financial relationships that could be construed as a potential conflict of interest.

## Footnotes

^1^The term Health Navigator is intended to be a generic term to describe the academic certificate or degree program. It is not designed as a job title and it is not intended to imply a connection with the Affordable Care Act.

^2^Association of State and Territorial Health Officials (ASTHO), National Association of County and City Health Officials (NACCHO), Society for Public Health Education (SOPHE), Association of University Programs in Health Administration (AUPHA), and the Association of Environmental Health Academic Programs (AEHAP).

## References

[1] Honore P Graham G Garcia J Morris M A call to action: public health and community college partnerships to educate the workforce and promote health equity. J Public Health Manag Pract (2008) 14(6):S82–4 10.1097/01.PHH.0000338392.90059.cd 18843245

[2] Kirkwood BA An Exploration of the Adoption of Public Health Degrees and Certificates in Community Colleges [Dissertation]. Ann Arbor: The George Washington University (2012). ProQuest/UMI (Publication No. 3503371.).

[3] American Public Health Association. Integration of Core Public Health Education into Undergraduate Curricula Policy Statement 200915. Available from: http://www.apha.org/policies-and-advocacy/public-health-policy-statements/policy-database/2014/07/22/09/58/the-integration-of-core-public-health-education-into-undergraduate-curricula

[4] Healthy People 2020 Objectives for 4-Year and 2-Year Colleges to Increase Public Health Education – See Public Health Infrastructure Topic Areas. Available from: http://healthypeople.gov/2020/topicsobjectives2020/objectiveslist.aspx?topicId=35

[5] League for Innovation in the Community College. Community Colleges and Public Health Project. Available from: http://www.league.org/league/projects/ccph/

[6] Association of Schools and Programs of Public Health. Framing the Future Task Force. Available from: http://www.aspph.org/educate/#educational-models

[7] Association of American Colleges and Universities. LEAP Initiative. Available from: https://www.aacu.org/leap/

[8] Association of American Colleges and Universities. VALUE Rubrics. (2014). Available from: http://www.aacu.org/VALUE/rubrics/

[9] Association of Schools and Programs of Public Health. Undergraduate Public Health Learning Outcomes. (2014). Available from: http://www.aspph.org/educate/models/undergraduate-learning-outcomes/

[10] Association of Schools and Programs of Public Health. Undergraduate Baccalaureate Critical Component Elements Report. (2014). Available from: http://www.aspph.org/educate/models/undergraduate-baccalaureate-cce-report/

[11] U.S. Bureau of Labor Statistics. Occupational Outlook Handbook. (2014). Available from: http://www.bls.gov/ooh/community-and-social-service/health-educators.htm

